# Causative classification of river flood events

**DOI:** 10.1002/wat2.1353

**Published:** 2019-05-26

**Authors:** Larisa Tarasova, Ralf Merz, Andrea Kiss, Stefano Basso, Günter Blöschl, Bruno Merz, Alberto Viglione, Stefan Plötner, Björn Guse, Andreas Schumann, Svenja Fischer, Bodo Ahrens, Faizan Anwar, András Bárdossy, Philipp Bühler, Uwe Haberlandt, Heidi Kreibich, Amelie Krug, David Lun, Hannes Müller‐Thomy, Ross Pidoto, Cristina Primo, Jochen Seidel, Sergiy Vorogushyn, Luzie Wietzke

**Affiliations:** ^1^ Department Catchment Hydrology Helmholtz Centre for Environmental Research Halle (Saale) Germany; ^2^ Institute of Hydraulic Engineering and Water Resources Management Vienna University of Technology Vienna Austria; ^3^ Helmholtz Centre Potsdam GFZ German Research Centre for Geosciences Potsdam Germany; ^4^ Institute for Environmental Sciences and Geography University Potsdam Potsdam Germany; ^5^ Department of Environment, Land and Infrastructure Engineering Politecnico di Torino Turin Italy; ^6^ Institute for Hydrology and Water Resources Management Leibniz University Hannover Hannover Germany; ^7^ Institute of Hydrology, Water Resources Management and Environmental Engineering Ruhr University Bochum Bochum Germany; ^8^ Institute for Atmospheric and Environmental Science Goethe University Frankfurt Frankfurt Germany; ^9^ Institute for Modelling Hydraulic and Environmental Systems University of Stuttgart Stuttgart Germany

**Keywords:** flood genesis, flood mechanisms, flood typology, historical floods, hydroclimatology of floods

## Abstract

A wide variety of processes controls the time of occurrence, duration, extent, and severity of river floods. Classifying flood events by their causative processes may assist in enhancing the accuracy of local and regional flood frequency estimates and support the detection and interpretation of any changes in flood occurrence and magnitudes. This paper provides a critical review of existing causative classifications of instrumental and preinstrumental series of flood events, discusses their validity and applications, and identifies opportunities for moving toward more comprehensive approaches. So far no unified definition of causative mechanisms of flood events exists. Existing frameworks for classification of instrumental and preinstrumental series of flood events adopt different perspectives: hydroclimatic (large‐scale circulation patterns and atmospheric state at the time of the event), hydrological (catchment scale precipitation patterns and antecedent catchment state), and hydrograph‐based (indirectly considering generating mechanisms through their effects on hydrograph characteristics). All of these approaches intend to capture the flood generating mechanisms and are useful for characterizing the flood processes at various spatial and temporal scales. However, uncertainty analyses with respect to indicators, classification methods, and data to assess the robustness of the classification are rarely performed which limits the transferability across different geographic regions. It is argued that more rigorous testing is needed. There are opportunities for extending classification methods to include indicators of space–time dynamics of rainfall, antecedent wetness, and routing effects, which will make the classification schemes even more useful for understanding and estimating floods.

This article is categorized under:

Science of Water > Water Extremes
Science of Water > Hydrological Processes
Science of Water > Methods

## 1 INTRODUCTION

River flood events exhibit a wide variety of process controls that determine their time of occurrence, duration, extent, and severity. However, generation mechanisms of river floods are not well defined at the catchment scale. As a consequence, the different generating mechanisms and characteristics of floods are usually ignored in statistical or comparative analyses. In fact, a fundamental hypothesis of extreme value statistics is that observations are homogenous and subject to a common set of forces (Gumbel, 1941), while flood event discharges observed in a catchment might be nonidentically distributed (because of their different origins) and nonstationary (due to the natural climatic variability and human interventions) (Hirschboeck, 1987; Merz et al., 2014). Collectively analyzing floods that are caused by different processes may result in uncertain predictions of flood characteristics (Potter, 1958) and their possible changes (Hirschboeck, Ely, & Maddox, 2000). For this reason, it is of advantage to only consider events that exhibit similar traits when comparing river floods in different periods or catchments (Blöschl, Sivapalan, Wagener, Viglione, & Savenije, 2013).

A consistent causative classification of flood events can help address the above‐mentioned issues (Blöschl, 2006), and contribute to a better process understanding of flood generation. Its use in flood statistics may improve at‐site and regional flood frequency estimates (Alila & Mtiraoui, 2002; Merz & Blöschl, 2008a, 2008b). A causative classification is also needed to identify and quantify changes in flood generating mechanisms (Hirschboeck et al., 2000; Nied et al., 2014) and assists in deciphering changes even if clear trends in climatic forcing or catchment conditions are not observed (Hirschboeck, 2009; Keller, Rössler, Martius, & Weingartner, 2018). Moreover, it would allow for evaluating whether a particular flood type has become more frequent over a number of decades instead of merely examining the magnitudes of all floods in a lumped way.

In this study, we review causative classifications of naturally occurring river floods and do not consider events caused by structure failures (e.g., dam breaches). The existing classification schemes can be grouped into three main categories according to the perspective they adopt for determining flood generating mechanisms: hydroclimatic, hydrological, and hydrograph‐based. The latter perspective does not focus on the generating mechanisms per se but on their effects on the hydrograph. However, we still consider these classifications when hypotheses on the links between effects and causative mechanisms are provided (Section 2.3). Similarly, we consider season‐based classifications as belonging to the hydrological category (Section 2.2.1) only if the day of year the event occurred (i.e., season) is used as a proxy for flood generating mechanisms (e.g., snowmelt or rainfall).

In any of these perspectives, flood types can be either predefined (deductive approaches, used when relevant processes are possibly known) or derived by data mining (inductive approaches, adopted when no specific mechanisms are assumed a priori). Both approaches aim to transfer multivariate metrics of the input data (i.e., characteristics or indicators) to univariate outputs (i.e., flood types) that represent specific generation mechanisms of floods.

The goal of this paper is to review existing causative classifications for instrumental and preinstrumental series of flood events and critically assess their validity, limitations, and transferability across temporal and spatial scales. In the first part, we review publications on causative classification of river flood events and their applications for various hydrological purposes. We examine the issues of data uncertainty, indicators, and methods selection on classification results. We further discuss testing and evaluation strategies that can assure practical applicability and transferability of the developed frameworks. Finally, we make recommendations for the further development and implementation of flood classifications, and discuss perspectives for moving from task‐specific and locally developed to more comprehensive frameworks.

## 2 CAUSATIVE CLASSIFICATIONS OF INSTRUMENTAL SERIES OF RIVER FLOOD EVENTS

**Figure 1:**
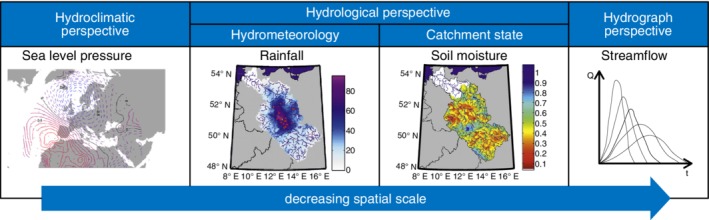
Different perspectives and scales of existing causative classifications of river flood events: Regional to subcontinental scale of hydroclimatic classifications; catchment to regional scale of hydrological perspective based on hydrometeorological forcing related to catchment and catchment state; and catchment scale of hydrograph‐based classifications. (Reprinted with permission from Bárdossy and Pegram (2011). Copyright 2011 Wiley and Nied et al. (2014). Copyright 2014 CC BY)

As early as 1930, Hazen (1930) noted that floods can be caused either by snowmelt, large wide storms, or local cloud bursts. Later, defining hydroclimatic or hydrometeorological contexts of floods became the foundation of causative classification of river flood events (Hirschboeck et al., 2000). The hydroclimatic context is represented by large‐scale or synoptic patterns of atmospheric pressure at the time an event occurred (Shelton, 2008) (Figure 1). Hydroclimatic classifications of floods originate from climatic transition zones where great differences among event hydrographs generated by different climatic and synoptic features were noticed.

The hydrometeorological context instead focuses on a specific location, that is, catchment, and describes a day‐to‐day state of the atmosphere, that is, variations of precipitation, temperature, wind, and other variables (House & Hirschboeck, 1997) for a certain event. When linked with catchment state characteristics (e.g., pre‐event soil moisture, snow depth), it can provide a comprehensive picture of the flood generation mechanisms at catchment and regional scale from a hydrological point of view. We will refer to this joint perspective as the hydrological perspective as, in this case, catchments are seen as elements which integrate all aspects of the hydrologic cycle within a defined area (Wagener, Sivapalan, Troch, & Woods, 2007) (Figure 1). Classifications from this perspective originate mostly from regions with cold and temperate climate, where the seasonality of the runoff regime and the differences between snowmelt and rainfall‐induced flood hydrographs are especially pronounced (Diehl & Potter, 1987).

Alternatively, classifications can adopt a hydrograph perspective and group flood events based on their effects (i.e., hydrographs; Figure 1), assuming that distinct effects result from different generating mechanisms. Approaches adopting this perspective emerged as a parsimonious method to improve the at‐site fit of flood frequency curves to observed flood samples (Singh, 1968).

In the next subsections, we review existing causative classifications of river flood events according to these three perspectives. Furthermore, we illustrate the difference between existing frameworks based on the example of classifications with hydrological perspective (Section 2.4).

### 2.1 Hydroclimatic perspective

Hydroclimatic classifications adopt a large synoptic domain and usually neglect the catchment state (Figure 1). These approaches focus on lifting mechanisms of moisture, its transport, weather systems, storm morphology, circulation patterns, and large‐scale climatic associations (Hirschboeck, 1988). The idea of hydroclimatically distinct events in flood series emerged from research on storm types and their characteristics (i.e., duration and intensity). One of the first classification proposed the existence of cold, warm and stationary fronts, squall lines, and storm events characterized by warm or cold air masses in Illinois, USA (Hiser, 1956). Essenwanger (1960) instead suggested considering hurricanes, extratropical cyclones, and frontal rains as distinct storm types.

The meteorological and climatic causes and preconditions of floods were further analyzed by Ward (1978), Maddox, Chappell, and Hoxit (1979), and Doswell, Brooks, and Maddox (1996). Analysis of individual extreme floods showed the exceptional importance of anomalous large‐scale atmospheric circulation patterns (e.g., blocking situations, atypical locations of synoptic features, rare combinations of atmospheric processes, and unusual configurations of circulation patterns) for generating catastrophic events (Blöschl, Nester, Komma, Parajka, & Perdigão, 2013; Grams, Binder, Pfahl, Piaget, & Wernli, 2014; Hirschboeck, 1987a).

Starting with the work of Canterford and Pierrehumbert (1977), a value of explicitly linking series of flood events to their hydroclimatic causes rather than considering only individual events was recognized. In the following subsections, we review such attempts to classify flood series.

#### 2.1.1 Hydroclimatically predefined types: Lifting mechanisms and weather systems

**Figure 2:**
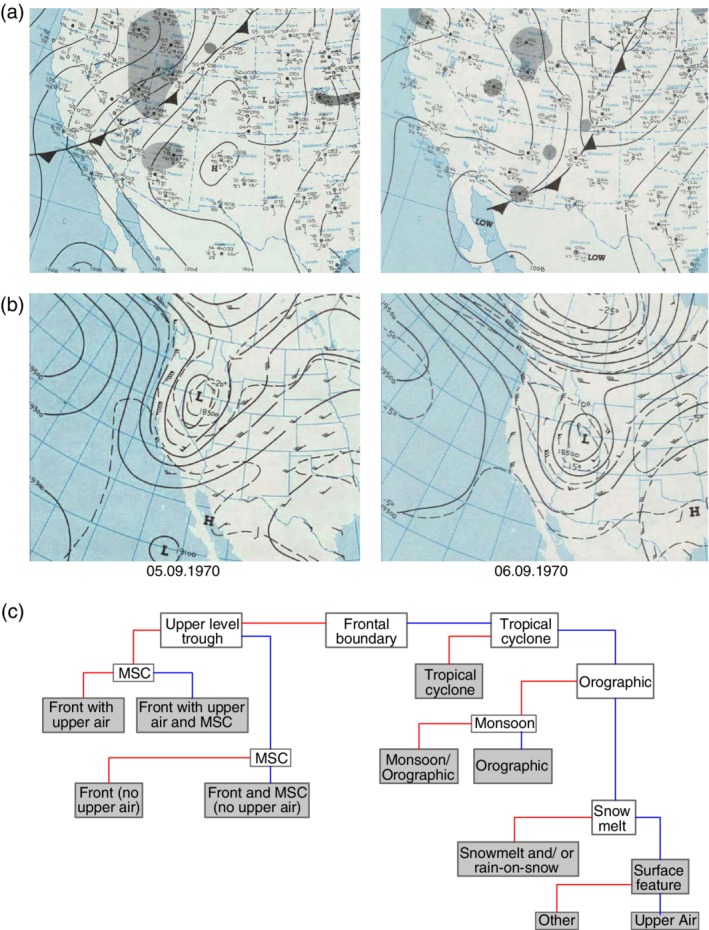
(a) Surface weather maps and (b) corresponding 500 hPa charts (NOAA Central Library Data Imaging Project, https://library.noaa.gov/Collections/Digital‐Collections/US‐Daily‐Weather‐Maps) for September 5–6, 1970 representing hydroclimatic sequence which produced widespread flooding in the Gila River Basin, Arizona (Hirschboeck, 1987); and (c) flow chart for determining synoptic and mesoscale environment (MCS—mesoscale convective system; upper air—upper‐level trough or closed low system) corresponding to flood events in contiguous USA. White boxes represent atmospheric features and factors used for classification; gray boxes represent determined flood type. Red and blue lines correspond, respectively, to presence and absence of certain feature. (Panel c Reprinted with permission from Ashley and Ashley (2008). Copyright 2007 Wiley)

These classifications stratify floods according to predefined types based on the structure and dynamics of weather systems and lifting mechanisms (e.g., extratropical cyclones and their embedded mesoscale fronts, tropical cyclones, mesoscale convective systems, orographic lifting) triggering rainfall events (see Table 1). The first comprehensive attempt to identify homogeneous subsets of floods produced by distinct weather systems was developed based on flood peak records of 30 gauges located in multiple subcatchments of the Gila River Basin which encompasses most of central and southern Arizona (Hirschboeck, 1985, 1987). Day of occurrence (i.e., season) is first used to discriminate floods produced during the Arizona summer convective storm season (also referred as the North American Monsoon). The presence of major synoptic features (tropical cyclones, cutoff lows, or fronts) on surface weather maps is then used to further stratify flood series (Figure 2a). Based on the spatial patterns of precipitation in the catchment, events are classified as widespread or localized. The latter are considered the product of local (convective) weather patterns, while the former indicates presence of a widespread synoptic situation. Finally, snowmelt flood events are additionally accounted for. Therefore, this classification combines lifting mechanisms, weather systems, and some degree of hydrological perspective into one framework.

**Table 1:** Existing causative classifications of river flood events: Hydroclimatic perspective based on lifting mechanism and weather systems (Section 2.1.1)^a^

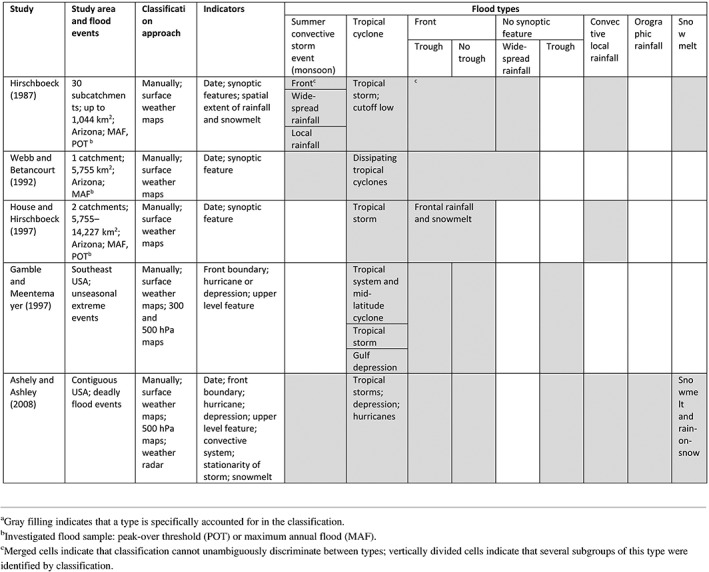

Simplified versions of this method, required by data constrains, were later adopted for several applications in the same basin identifying each only three major flood types: monsoons, frontal systems, and dissipating cyclones (Webb & Betancourt, 1992); or tropical cyclones, fronts, and convective storms (House & Hirschboeck, 1997). These revised classifications are purely based on lifting mechanisms and weather systems, with no hydrological insight.

Classifications of flood events based on lifting mechanisms were further developed by Gamble and Meentemeyer (1997) and Ashley and Ashley (2008). Additionally to considering cold or warm fronts associated with mid‐latitude cyclones and tropical cyclones identified on surface weather maps (Figure 2a) they accounted for upper level enhancement (troughs) analyzing 500 hPa geopotential height maps (Figure 2b). Based on weather radar data information on the existence of mesoscale convective systems or orographic enhancement (stationary thunderstorms) is also included (Table 1, Figure 2c). Their comprehensive approach extended the existing hydroclimatic classifications beyond the Southwest to the contiguous United States.

#### 2.1.2 Atmospheric circulation patterns, cyclone tracks, and moisture transport as hydroclimatic types

Another hydroclimatic approach to classify floods links the occurrence of particular events with that of certain atmospheric circulation pattern, cyclone track, or low‐frequency climate variability. When performed for series of floods, it can provide essential information about their causes.

The analysis of long‐term climate variations (e.g., Southern Oscillation Index, Pacific Decadal Oscillation) provides indications about nonstationarity in the flood sample (Villarini, Smith, Serinaldi, Ntelekos, & Schwarz, 2012; Webb & Betancourt, 1992). The link with floods is usually established by the year of flood occurrence (Alila & Mtiraoui, 2002) or by constructing Poisson regressions between the frequency of peak‐over‐threshold (POT) flood events and climate indices (Mallakpour & Villarini, 2016).

At shorter time scales, synoptic climatology provides an opportunity to link categorized climatic and weather systems with the events observed at regional or local scales (Yarnal, 1993). Concrete evidences of a relationship between circulation patterns and hydrometeorological observations (Bárdossy & Caspary, 1990) support the use of circulation classifications as the basis for classifying causation in flood series. In this way, main flood‐inducing circulation patterns, their role in the seasonality of flood regimes and the occurrence of floods of certain return periods can be identified (Petrow, Merz, Lindenschmidt, & Thieken, 2007). It also provides an opportunity to link detected flood changes to changes of prevailing circulation patterns (Frei, Davies, Gurtz, & Schär, 2000; Petrow, Zimmer, & Merz, 2009) or in the large‐scale climatic boundary conditions (Delgado, Merz, & Apel, 2012).

Classification of circulation patterns can be subjective (based on expert knowledge about the effect of certain circulation types on surface climate parameters), threshold‐based (defining types by setting thresholds between them), or based on clustering of sea level pressure and geopotential heights (Philipp et al., 2010). The addition of zonal and meridional wind components, precipitable water, and temperature information allows for classification of weather pattern types (Murawski, Bürger, Vorogushyn, & Merz, 2016).

Classifications of circulation patterns have either predefined or derived types, which correspond to different views of synoptic climatology: “circulation‐to‐environment” or “environment‐to‐circulation” (Yarnal, 1993). The latter perspective classifies atmospheric states when specific event occur and provides a greater insights about event origin (Lee & Sheridan, 2015). In this case, classification methods based on optimizing objective functions can be used to identify specific flood‐inducing circulation patterns (i.e., patterns corresponding to high discharge increments, Bárdossy & Filiz, 2005). The former perspective offers a more general but well‐documented daily catalogues (e.g., Hess & Brezowsky, 1977; Lamb, 1950). However, due to the delay between the occurrence of patterns and catchment responses, they cannot be directly linked. It can be overcome by considering a mean catchment concentration time or examining different lag times (Duckstein, Bárdossy, & Bogárdi, 1993; Petrow et al., 2009).

Daily classification of circulation patterns might be insufficient for representing the full complexity of evolving atmospheric situations. For this reason, sequential classification (compound types of up to four sequential daily circulation patterns) can be applied to characterize build‐up processes of each event (Yarnal & Frakes, 1997).

Hydroclimatic stratification of flood events can be also performed using spatiotemporal rainfall fields prior to flood events as an input for clustering. This approach was applied to the large Upper Parana River Basin, Brazil (Lima, AghaKouchak, & Lall, 2017). Based on distinct rainfall patterns, four flood types related with different atmospheric circulation and moisture transport were derived that were associated with tropical and extratropical processes, such as extratropical cyclones, South Atlantic Convergence Zone, South America low‐level jet, as well as distinct patterns of sea surface temperature.

Another promising alternative is the attribution of floods to cyclone track types. Cyclone tracks are classified based on the geographic regions crossed before reaching the target region, thus providing information on moisture sources (Hofstätter, Chimani, Lexer, & Blöschl, 2016), the origin of flood‐producing storms, their evolution in time and space (Collins et al., 2014; Hofstätter, Lexer, Homann, & Blöschl, 2018), and eventually on synchronization of flood events at larger scales.

### 2.2 Hydrological perspective: Hydrometeorological forcing and catchment state

This section analyses causative classifications of flood events based on hydrometeorological variables (e.g., rainfall, temperature) observed within catchments, the catchment state (e.g., snow depth, soil moisture), and hydrological processes (e.g., infiltration or saturation excess) leading to floods. These three approaches are considered together because they adopt the same scales (from catchment to regional) (Figure 1), their indicators usually refer to specific catchments and they attempt to characterize flood events from the hydrologic point of view. Early classifications focused on seasonality of hydrological processes and essentially distinguished rainfall and snowmelt‐induced floods (Section 2.2.1; Table 2a). Similar to the first hydroclimatic classifications, they were developed to improve at‐site flood frequency analysis. Later on, a wide range of complex multicriteria classifications shaped by local and regional conditions were proposed to identify catchment‐scale flood generation mechanisms (Sections 2.2.2 and 2.2.3, Table 2b). Attempts to link the hydrological classification perspective to the occurrence of distinct atmospheric circulation patterns (Nied et al., 2014; Nied, Schröter, Lüdtke, Nguyen, & Merz, 2017) or cyclone tracks (Collins et al., 2014) also exist, but a comprehensive framework that merges both perspectives has not been developed.

**Table 2:** Existing causative classifications of river flood events: Hydrological and hydrograph‐based perspectives^a^

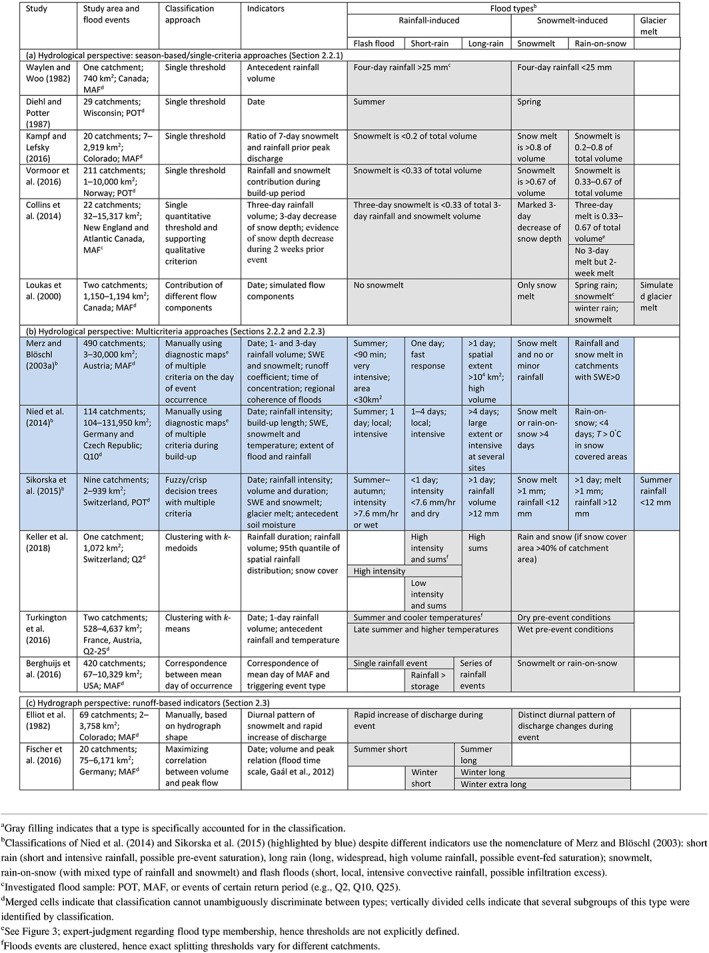

#### 2.2.1 Season‐based approaches

Approaches discussed in this section adopt seasonality as a proxy for distinct flood generation mechanisms (Table 2a). This method was developed to identify flood samples in mid‐latitude river basins characterized by pronounced seasonal differences in their flood regimes. Starting with the work of Todorovic and Rousselle (1971) in the Greenbrier River (West Virginia, USA), the date of flood occurrence was often used as a simple criterion to classify floods (e.g., Browzin, Baumbusch, & Pavlides, 1973; Guillot, 1973). Later on, Diehl and Potter (1987) identified two seasonal populations of floods in Wisconsin, USA, arguing that spring floods differ from summer floods due to snowmelt and no infiltration on frozen soils. However, already Gupta, Duckstein, and Peebles (1976) voiced concerns about the physical arbitrariness of dividing observations into seasonal intervals, as different types can occur in the same season, especially in climatically diverse regions. Analyzing Italian floods, also Rossi, Fiorentino, and Versace (1984) concluded that the assumption of identically distributed flood peaks within a season is not realistic. Despite these criticisms, seasonality was until recently used as sole criterion for causative classification of river floods (e.g., Kochanek, Strupczewski, & Bogdanowicz, 2012; Merz, Piock‐Ellena, Blöschl, & Gutknecht, 1999; Singh, Wang, & Zhang, 2005), even in model‐based classifications (e.g., Loukas et al., 2000).

In order to strengthen the physical bases of flood event stratification, Waylen and Woo (1982) distinguished rainfall and snowmelt generated floods using a 4‐day antecedent rainfall index in a mesoscale Canadian catchment in the Cascade Mountains. However, the selected indicator is essentially a substitute for the day of occurrence, and assumes that in spring and summer all flood events are generated by snowmelt, while in autumn and winter they are caused by rainfall. Later, Vormoor, Lawrence, Heistermann, and Bronstert (2015) and Kampf and Lefsky (2016) discriminated snowmelt, rainfall, and mixed floods based on the volumetric ratio between simulated snowmelt and observed rainfall. Collins et al. (2014) classified flood events into four generation mechanisms (rainfall, snowmelt, rain‐on‐snow, and rainfall/snowmelt) using observed precipitation and snow depth. Instead of using volumetric ratio as single indicator, they additionally used a qualitative criterion of snowmelt occurrence within 2 weeks prior to the event. However, other distinctions than the one between snowmelt and rainfall‐induced floods are important. In fact, floods exhibit varied characteristics also when rainfall is produced by different mechanisms (Maddox et al., 1979). In the following two sections, we review multicriteria classifications with predefined and derived flood types that move beyond seasonal classifications and consider flood generation mechanisms at catchment or regional scales by using hydrometeorological variables observed in catchments and indicators of catchment states as the basis for classification.

#### 2.2.2 Multicriteria approaches: Hydrologically predefined types

**Figure 3:**
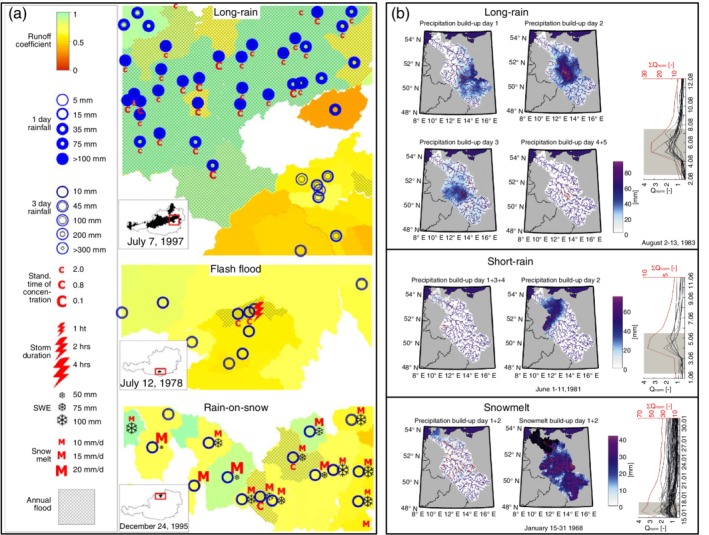
An example of diagnostic maps for classification of river flood events based on expert judgment according to (a) Merz and Blöschl (2003): Diagnostic maps of event‐ and catchment‐averaged indicators (event runoff coefficient, 1‐day and 3‐day rainfall depth, standardized time of concentration, storm duration for documented convective thunderstorms, snow water equivalent [SWE], snowmelt amount) on the day of occurrence of various annual flood events in Austria. (Reprinted with permission from Merz and Blöschl (2003). Copyright 2003 Wiley); (b) Nied et al. (2014): Daily diagnostic maps of build‐up period for exemplarily flood events in the Elbe catchment. The intensity of blue color indicates daily precipitation or snowmelt amount [mm]. Affected gauges are indicated by the red dots. The hydrographs (black lines) in the left panel correspond to observed discharge at the affected gauges normalized by their 2‐year flood. Red line corresponds to the discharge sum. Gray rectangular indicates the build‐up period of flood event. (Reprinted with permission from Nied et al. (2014). Copyright 2014 CC BY)

Causative classifications listed in this section were developed for understanding catchment‐scale flood generation mechanisms and their regional differences. In order to identify these mechanisms, multicriteria approaches that consider a broad range of hydrometeorological and hydrological data and indicators are required. The first classification of this kind, proposed by Merz and Blöschl (2003) and developed for a large set of Austrian catchments, includes five types of river flood events (short rain, long rain, snowmelt, rain‐on‐snow, and flash floods) characterized by indicators derived from hydrometeorological observations and modeled catchment states (Table 2b, Figure 3a). In this context, flash floods are events triggered by very short and intensive rainfall of mostly convective origin and generated locally by infiltration excess mechanisms. Short‐rain floods are also produced by local and intensive rainfall, but with longer duration and weaker intensity. Generation of this type can be enhanced by wet antecedent conditions. Long‐rain floods are instead generated by partial or full catchment saturation as a result of widespread long rainfall with low intensity. Rain‐on‐snow floods are triggered by rainfall on snow‐covered areas, while snowmelt floods are caused by melting of accumulated snow due to temperature increase. This classification was later adopted (although using different indicators, see Section 2.4) for subcatchments of the Elbe River (Nied et al., 2014) and for Switzerland, by adding glacier melt floods to the original flood types (Diezig & Weingartner, 2007; Sikorska et al., 2015).

An alternative classification of flood generation mechanisms was suggested by Berghuijs et al. (2016) for the contiguous United States. It distinguishes four types of floods, generated by single large rainfall events, series of rainfall events, precipitation excesses, and snowmelt or rain‐on‐snow events, respectively. Their approach does not classify every recorded flood, but rather searches for overlapping in the day of occurrence between floods and the four predefined types of triggering events. It can thus be used to test hypotheses regarding dominant flood generation processes and their role in triggering extreme floods. Their results suggest a weak effect of extreme precipitation and a relevant role of catchment storage (antecedent wetness state). Therefore, soil moisture appears to be an important factor that is currently underrepresented as indicator in the reviewed classifications (Table 2).

#### 2.2.3 Multicriteria approaches: Hydrologically derived types

Approaches of this family use clustering methods to group flood events and derive flood types through inductive analyses. Types do not have to be defined a priori, but relevant indicators and the number of clusters should be selected based on expert judgment or assessing their values by means of statistical measures of clustering performance.

**Figure 4:**
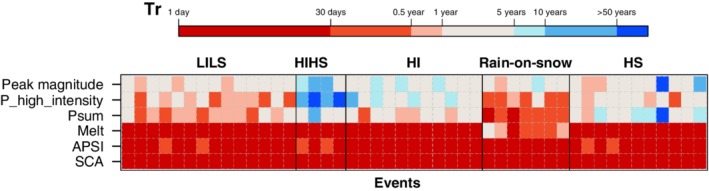
Empirical return periods (Tr) of parameter event values for different flood‐precursor storylines (i.e., hydrometeorological and catchment state conditions leading to flood event): LILS low intensity—low sums; HIHS high intensity—high sums; HI high intensity; rain‐on‐snow; HS high sums. Each row corresponds to return period of respective parameter: APSI—antecedent precipitation and snowmelt index; SCA—snow‐covered area; Psum—volume of rainfall; P_high_intensity—95th quantile of spatial precipitation distribution on the day with maximum rainfall amount; melt—accumulated snowmelt over the course of the event; peak magnitude—Peak discharge of respective flood event. Each column corresponds to an individual flood event. (Reprinted with permission from Keller et al. (2018). Copyright 2017 Wiley)

Keller et al. (2018) clustered flood events in a mesoscale Swiss catchment and identified five distinct flood‐precursor storylines (i.e., causal chain of hydrometeorological factors and catchment state conditions leading to a flood event) that are to some extent comparable to the flood types of Merz and Blöschl (2003) (Section 2.2.2). The “High Sums” cluster, which comprises floods with long duration and low intensity, resembles the long‐rain flood type. The “High intensity” cluster groups flash flood events, even if it cannot be directly compared to this type as only daily hydrometeorological data are used by Keller et al. (2018). Floods belonging to the “Rain‐on‐snow” cluster are identified only considering simultaneity of rainfall and snow cover in the catchment in time. In contrast to other methods, the spatial distribution of precipitation (i.e., its 95th quantile in days with maximum precipitation intensity) is considered. This allows them to characterize episodes of low or high rainfall intensity embedded into longer events and to identify “Low intensity – low sums” and “High intensity – high sums” flood precursors that would be otherwise overlooked. The indicators and the empirical return periods of floods resulting from these storylines are clearly distinct from other clusters (Figure 4) highlighting the role of spatial characteristics of rainfall as an indicator of possible flood generating mechanisms.

The classification of Turkington et al. (2016) using derived flood types was explicitly developed to address their possible future changes resulting from climatic projections. This task requires the choice of simple flood type indicators (e.g., day of occurrence, 1‐day precipitation volume, antecedent precipitation, and temperature) which, however, do not unambiguously identify flood generating mechanisms (Table 2b). It is worth noting that using clustering methods to derive flood types inevitably results in the identification of different flood types for different catchments and depends on the considered flood sample.

### 2.3 Hydrograph perspective: Classification of effects

Hydrograph‐based classifications assume that different effects (i.e., hydrographs) reflect distinct mechanisms of flood generation. Their parsimony (they require no additional information except discharge time series) is an advantage for practical applications, but hydrograph characteristics alone might not be able to unambiguously identify processes responsible for flood generation (Table 2b and c). The ultimate objective of these methods is usually improving at‐site flood frequency analysis, hence derived flood types are tailored to specific locations.

A visual approach can be used for hydrograph‐based classifications. Elliott, Jarrett, and Ebling (1982) manually separated floods in the Colorado Front Range, USA into snowmelt and rainfall floods based on the shape of the hydrograph (i.e., visible diurnal patterns for snowmelt floods and rapid increase for rainfall floods). Daily and seasonal occurrence and local weather conditions were used then to check their plausibility (i.e., to validate that the effects actually represent the hypothesized causes).

**Figure 5:**
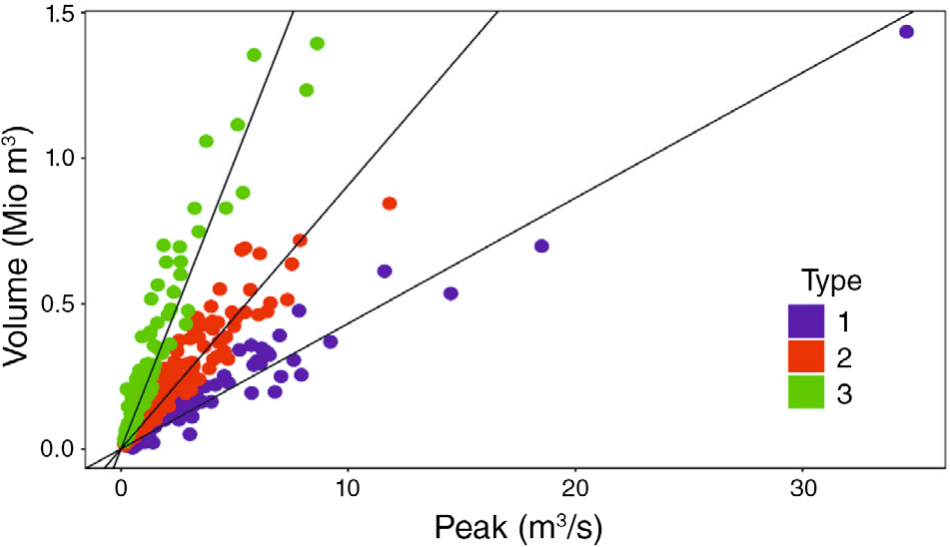
Distinction of flood events into different event types by maximizing correlation between event volume and peak discharge for the Holtemme River, Germany. Type 1, 2, and 3 correspond to short, long, and very long event time scales (Gaál et al., 2012). (Reprinted with permission from Fischer (2018). Copyright 2018 Taylor & Francis)

Intraseasonal flood types are derived by Fischer et al. (2016) using a measure of flood duration (i.e., the volume to peak ratio) adopted from Gaál et al. (2015). Based on their assumption that a weak correlation between volume and peak discharge is an indicator of different flood types in a seasonal sample, types are stratified by maximizing the correlation between volume, and peak flood discharge within subsamples (Figure 5). Their method applied to subcatchments of the Mulde River, Germany showed the presence of at least five different flood types in the sample of maximum annual floods (MAFs) (summer short, summer long, winter short, winter long, and winter extra‐long floods), which to some extent can be explained by different flood generating mechanisms (Table 2c).

### 2.4 Differences between frameworks—An example

Using the classifications of Merz and Blöschl (2003), Nied et al. (2014), and Sikorska et al. (2015) (Table 2b, rows highlighted by blue color), which propose the same causative flood types (Section 2.2.2), we illustrate how the choice of thresholds, classification methods, and input data affects the definitions of flood types and conceptualization of flood generation mechanisms.

The studies of Merz and Blöschl (2003) and Nied et al. (2014) focus on annual floods and regional floods with at least 10‐year return period respectively. They rely on expert judgment supported by either diagnostic maps of selected catchment‐ and event‐averaged indicators (Figure 3a) or daily diagnostic maps during build‐up periods that vary among events (Figure 3b), explicitly accounting for regional coherence of floods and storms. Sikorska et al. (2015) instead analyze POT events catchment‐wise, and employ conceptual threshold‐based decision trees for automated event classification.

Seasonality of events is considered in all three methods, but its indicator (i.e., date of occurrence) is used differently. Merz and Blöschl (2003) and Nied et al. (2014) use it to distinguish short rain from flash floods, assuming that convective processes causing the latter are only possible in summer. Sikorska et al. (2015) instead introduce seasonally varying indicators of flood types. Accordingly, an event with very high intensity and short duration occurring in the late autumn can be also classified as a flash flood (see Figure 2 of Sikorska et al., 2015).

All three methods use rainfall volume, intensity, and duration, but the values of splitting thresholds vary due to different temporal resolution of the input data, thus affecting the definitions of short rain, long rain, and flash floods. Differently from the other two methods, Nied et al. (2014) account for the space–time distribution of rainfall by analyzing daily gridded rainfall fields during build‐up periods (Figure 3b). Therefore, it allows for identifying two subtypes of long‐rain floods: basin‐wide low intensity rainfall and spatially limited high intensity rainfall (Table 2b).

The methods of Merz and Blöschl (2003) and Sikorska et al. (2015) account for catchment wetness. States prior to events, characterized by means of simulated soil moisture, are used in the latter study to distinguish short rain and flash floods (Table 2b). The former study instead implicitly considers catchment wetness in terms of the event runoff coefficient, which is used as an auxiliary indicator of runoff generation processes but not as a critical criterion for flood type assignment (see Table 1 of Merz & Blöschl, 2003). Differently from the other two studies, they consider runoff response dynamics, associating fast response to flash and short rain and slower response to long rain and snowmelt floods.

The definitions of rain‐on‐snow floods vary as well. Nied et al. (2014) explicitly account for spatial overlapping of rainfall and snow covered areas, whereas the other two studies only account for temporal overlapping. It results in a mixed class of simultaneous rain‐on‐snow, radiation‐induced snowmelt, and rainfall floods. It is worth stressing that simultaneously occurring rainfall and snowmelt events might have considerably different characteristics than rain‐on‐snow floods. In this latter case, runoff can indeed either be immediately released through preferential flow paths, thus determining severe floods, or be considerably delayed due to refreezing (Würzer et al., 2017).

Despite the usefulness of these classifications for the particular catchments/regions they were developed for, the above mentioned differences make comparison of their results rather difficult, and limit their transferability to other locations.

## 3 CAUSATIVE CLASSIFICATIONS OF PREINSTRUMENTAL SERIES OF RIVER FLOOD EVENTS

In reconstruction of historical flood events based on documentary evidence flood classification mostly refers to the severity of events and impact assessment (Brázdil et al., 2012; Glaser et al., 2010). However, if the sources are detailed, enough causative attribution can be performed as well. All three perspectives of causative classifications presented above (Section 2) are used in historical flood research.

Hydroclimatic perspective (Section 2.1) was adapted to link winter floods occurred after AD 1500 on four German rivers to large‐scale atmospheric circulations (Jacobeit, Glaser, Luterbacher, & Wanner, 2003). For the same period, summer and winter floods in southern Germany were grouped based on prevailing circulation patterns (Sturm et al., 2001). Winter and summer floods of past 500 years on the Elbe and Oder Rivers were related to zonal westerly airflow and to the Vb cyclonic pathway, respectively (Mudelsee, Börngen, Tetzlaff, & Grünewald, 2004). Spatial extent of floods was linked to main synoptic causes in the Iberian Peninsula by Barriendos and Rodrigo (2006). Using similar approach, Himmelsbach, Glaser, Schoenbein, Riemann, and Martin (2015) defined five types of specific spatial patterns of floods that affected the Rhine tributaries in Germany and France in the last 500 years.

Application of hydrological perspective (Section 2.2) for causative classification of floods in historical flood research is particularly related to season‐based approaches. These approaches are applied when the date, month, or season of the flood event is known with high certainty from systematic accounts (e.g., bridgemasters' accounts) or legal documentation (e.g., charters), but little or no certain information is available concerning its causes (Brázdil et al., 2014; Kiss, 2018; Rohr, 2007). Such approach was used for classification of historical floods in the Czech Lands into distinct winter and summer types (Brázdil et al., 2005). Historical flood investigations along the eastern Iberian Peninsula identified convectional atmospheric activity as the cause of local catastrophic summer and autumn floods. In the north also spring floods belonged to this group, while in the Ebro basin spring floods were attributed to snowmelt origin with additional intense rainfall (Barriendos & Vide, 1998).

More detailed multicriteria approaches of hydrological perspective are applied when additional information from contemporary documentation is available. However, even in these cases, there is more chance to find information on the hydrometeorological situation of a specific flood event than on catchment state. Most commonly used causative flood types are summarized in Table 3. Glaser et al. (2010) classified historical flood series in Central and Southern Europe as floods caused by convective rain, long‐lasting rain, snowmelt, torrential rain, and ice‐jam floods. The historical floods of the Rhône River in the last 800 years were classified into three causative types: ice jams, rainwater flooding, and sea intrusion (Pichard, Arnaud‐Fassetta, Moron, & Roucaute, 2017).

**Table 3:** Causative flood types of hydrological perspective frequently applied in historical flood research

Type of historical flood events	Local and regional studies
Flood caused by prolonged/heavy rainfall	Brázdil et al. (2005, 2011); Barrera, Barriendos, and Llasat (2005); Glaser et al. (2010); Retsö (2015)
Flash flood caused by torrential/incessant rains	Llasat, Barriendos, Barrera, and Rigo (2005); Barrera et al. (2005); Barrera, Llasat, and Barriendos (2006); Dolák, Brázdil, and Valášek (2013); Herget, Roggenkamp, and Krell (2014)
Snowmelt (thaw) flood	Mudelsee, Deutsch, Börngen, and Tetzlaff (2006); Brázdil et al. (2011); Retsö (2015)
Flood caused by ice jamming	Rohr (2007); Brázdil et al. (2010); Kiss (2007)

There are large local and regional variations in detected causative flood types and subtypes. In Scandinavia, special ice‐related causative categories exist, including ice run and jam. During ice run flood, the ice is not necessarily blocked, but by its quantity, ice is the main cause of flooding (Roald, 2013). In Central Europe, this flood type also appears in historical documentation as ice flow flood or is combined with ice‐jam floods. Although rarely observed nowadays, ice‐jam floods appear particularly often in historical flood chronologies in Central Europe (Brázdil et al., 2005; Kiss, 2018; Rohr, 2007).

Another, well‐defined flood type in historical flood investigations is flash flood. Analysis of rainfall duration and catchment response characteristics of historical flood events indicated that flash floods are responsible for most catastrophic flooding in Barcelona, Spain from the late Middle Ages onwards (Barrera et al., 2005). Later, flash floods were further stratified by intensity, duration, and spatial extension of the triggering rainfall events from local short‐lived to slightly convective events with long duration developed in catchments with area over 1,000 km^2^ (Barrera et al., 2006; Llasat et al., 2005). Furthermore, flash floods caused by torrential or incessant rains particularly often appear in taxation records and form the basis for flash flood reconstructions in the Czech Lands (Dolák et al., 2013). In South Moravia “ordinary” floods, flash floods, and inundation of fields and meadows were differentiated in historical taxation records as well (Brázdil et al., 2014).

The hydroclimatic and hydrological perspectives are often combined in studies on extraordinary large‐scale flood events or their series: the 1432 flood event in Bohemia (Brázdil, Kotyza, & Dobrovolný, 2006); the greatest floods on the Upper‐Rhine including the 1480 event (Pfister & Wetter, 2011; Wetter et al., 2011); the 1617 flood in Spain (Thorndycraft, Barriendos, Benito, Rico, & Casas, 2006); the July 1342 flood in Germany (Herget et al., 2014); and the series of events in Europe in 1783–1784 (Brázdil et al., 2010).

Finally, a perspective similar to hydrograph‐based approaches (Section 2.3) can be used in special cases when the cause of a flood event is not documented, but the information that resembles the evidence gained from daily flood curve might be available in contemporary sources (e.g., detailed eye‐witness reports, Kiss, 2018) and can facilitate attribution of the cause.

There is also a potential for the development and application of causative classifications in reconstruction of preinstrumental flood series based on indirect evidence in the paleorecord derived from geomorphic, botanical, and lichenometric indicators (Baker, 1998). Due to high temporal and spatial resolution, tree rings and sedimentary records might be a source of paleoflood information for linking reconstructed flood records to their hydroclimatic and hydrometeorological causes in the environments with good preservation conditions (Ballesteros‐Cánovas, Stoffel, St George, & Hirschboeck, 2015; Schulte et al., 2015). Understanding the causes of past floods reconstructed based on documentary evidence and paleorecords might provide relevant information for flood risk assessment under possible changes.

## 4 ROBUSTNESS CHECK AND UNCERTAINTY ANALYSIS

The example in Section 2.4 illustrates how results of causative classifications depend on the choice of input data and relevant indicators. Classification results are therefore sensitive to the uncertainty of input data from different sources. Kampf and Lefsky (2016) showed that utilizing two different temperature datasets considerably affects classification of snowmelt and rainfall‐induced floods. The same might be true for precipitation datasets. Unfortunately, robustness checks and uncertainty analyses lack in most studies on causative classification of flood events. This is often justified with the limited availability of data sources. However, if several datasets of the input variables are available, the clustering sensitivity to input data should be examined to assure robust results.

The most common causative classification relates to a distinction between snowmelt and rainfall floods. To this end, snowmelt is usually computed by calibrated hydrological models (Loukas et al., 2000; Merz & Blöschl, 2003; Sikorska et al., 2015) or simple degree‐day methods (e.g., Berghuijs et al., 2016; Brunner et al., 2017; Vormoor et al., 2016). Snowmelt estimates obtained from hydrological models calibrated only on discharge time series involve large uncertainties (Parajka & Blöschl, 2008; Tarasova, Knoche, Dietrich, & Merz, 2016). Therefore, also the uncertainty of the model (i.e., parameter settings, process implementation, selected input data, and calibration techniques) should be accounted for.

Using several data types to describe the same processes can improve robustness of threshold‐based classifications and assist in uncertainty attribution (Razavi & Gupta, 2015). For example, melting processes can be described by modeled snowmelt or alternatively by station or satellite‐observed snow cover (Keller et al., 2018). Similarly, catchment wetness can be represented by modeled soil moisture (Sikorska et al., 2015), observed antecedent precipitation integrated over different time periods (Turkington et al., 2016), or precipitation indices (Brunner et al., 2017; Keller et al., 2018). However, the usage of highly correlated variables for clustering might affect its performance. This issue can be addressed by applying principal component analysis (Mardia, Kent, & Bibby, 1979), which merges all the explanatory variables into a few uncorrelated components. Another option is selecting relevant variables that lead to the largest difference among clusters (Keller et al., 2018). However, it does not guarantee that the selected variables are representative indicators of flood generating mechanisms.

The sensitivity of the classification results to the classification thresholds is another issue for both single and multicriteria approaches. Crisp (sharp) thresholds are very sensitive to noise that might originate from the inconsistency of observed input data, uncertainties of the modeled data and inappropriate choice of thresholds (Sikorska et al., 2015). The uncertainty of splitting thresholds can be accounted for by fuzzy classification approaches (e.g., fuzzy decision trees, Sikorska et al., 2015) that use soft thresholds defined as a range of values, which are linked to specific degrees of membership of different flood origins. In this way, each flood can have mixed origin and the one with the highest degree of membership is regarded as the dominant flood type. The robustness of classifications based on conceptual decision trees with respect to threshold changes can be assessed in Monte Carlo experiment where thresholds are represented as random variables with assumed probability density function. Random sampling provides a new value for each tree threshold that is subsequently used for classification. Fuzzy decision trees were found to be more robust (i.e., less sensitive to changes in assigned thresholds) that crisp trees for causative classification of flood events (Sikorska et al., 2015). For applications, however, crisp thresholds may be preferable (Brunner et al., 2017). Development of continuous dimensionless indices instead of discrete flood classes, similar to those used in fluid mechanics (e.g., Reynolds and Froude numbers; Munson, Young, & Okiishi, 2002) to describe the character of flow, is another opportunity (Viglione, Chirico, Woods, & Blöschl, 2010).

## 5 TESTING AND EVALUATION OF CLASSIFICATION FRAMEWORKS

A drawback of existing classifications is a general lack of evaluating their accuracy for representing the hypothesized causative processes. Ideally, one would like to see evidence that the resulting subsamples of flood events are caused by distinct physical processes (Bardsley, 2016). Although this is rarely possible in practice, the classification ability to correctly identify causative mechanisms of flood event generation can be evaluated by comparing the results with detailed information about well‐observed flood events. Likewise, classifications that use coarse temporal and spatial data can be evaluated against the same frameworks applied to data with higher quality and resolution. Alternatively, synthetic experiments can be used to evaluate reliability of the proposed classifications.

Most causative classifications of flood events were developed to improve at‐site flood frequency analysis (see Section 6). Therefore, the causative homogeneity of the identified flood subsamples (i.e., the classification accuracy) was usually confirmed if the compound distribution was a better fit to the observed sample than the classical homogeneous distributions (e.g., generalized extreme value, Gumbel). Nevertheless, Bardsley (2016) asserted that a good fit of multicomponent distributions to the data does not imply good predictive performance especially if no explicit classification of flood events is performed.

Despite great methodological differences, almost every study reported a better fit of compound (flood type‐based) distributions compared to homogeneous distributions of unclassified floods. Thus, the question arises if improvements are due to causation‐informed homogeneity of flood subsamples or to the increased flexibility of compound distributions, which as a rule have more parameters (Fischer et al., 2016; Loukas et al., 2000; Singh, 1968; Singh et al., 2005). More rigorous testing involves comparing the performance of compound distributions with those of homogeneous distributions with the same number of parameters (Alila & Mtiraoui, 2002), or using information criteria (e.g., Akaike information criterion) that penalize distributions with a higher number of parameters.

Causative classifications are thought to improve the hydrological basis (i.e., improve robustness) of statistical models used for flood frequency analysis (Merz & Blöschl, 2008a). Therefore, the information value of flood types can be assessed by using longer observation records at the same site and testing the reliability of extrapolations from compound and homogeneous distributions to estimate floods with large return periods (Klemeš, 1986; Merz & Blöschl, 2003b). Alternatively, the value of flood type information can be also compared with random or hydrologically meaningless grouping.

Causative classifications that are only based on precursors' storylines (rainfall characteristics, antecedent soil moisture) and do not include any runoff characteristics can be evaluated by examining if different flood types exhibit distinct hydrograph characteristics (e.g., rise time, shape, and peak magnitude) (Berghuijs et al., 2016; Kampf & Lefsky, 2016; Keller et al., 2018). The hypothesized existence of distinct flood types can be also validated through statistical testing of the distribution equality of their event properties (Diehl & Potter, 1987).

## 6 APPLICATION OF CAUSATIVE CLASSIFICATIONS OF RIVER FLOOD EVENTS

Application of causative classifications of river flood events has a crucial role for strengthening the hydrological basis of flood estimation and prediction procedures and aids shifting from statistical flood frequency analysis to flood frequency hydrology (Merz & Blöschl, 2008a, 2008b; Viglione, Merz, Salinas, & Blöschl, 2013). However, their application is still mainly limited to derivation of flood‐type‐based compound distributions for improving at‐site flood frequency analysis (Hirschboeck et al., 2000).

Stratifying flood samples can benefit regional flood frequency analysis, either through the identification of homogeneous regions with same dominant flood generation mechanisms or the regionalization of flood‐type‐specific distribution parameters and moments. Physical characteristics of catchments (e.g., climate, soil type, and land use) are known to be poor regional predictors of flood moments (Merz & Blöschl, 2009). Flood‐type‐based regional flood frequency analysis might be a key to improve such estimates. For example, Jarrett and Costa (1988) examined regional differences of causative flood types in the Colorado Front Range, linked them to elevation zones and used this relationship to improve regional flood frequency predictions.

Analyzing spatiotemporal distribution of flood types can help adjust monitoring strategies for specific areas and flood genesis, highlight the importance of investigating certain generation processes (Würzer, Jonas, Wever, & Lehning, 2016), and define correct space–time scales to monitor rainfall in given catchments (Viglione, Chirico, Komma, et al., 2010). Finally, insights on people's preparedness to respond to the occurrence of specific flood types can be gained by linking flood generating mechanisms with records of deadly floods (Ashley & Ashley, 2008).

A recent study of Brunner et al. (2017) derived flood‐type specific synthetic hydrographs for the design of hydraulic structures and showed the importance of accounting for differences in the volume‐peak relationship of distinct flood types.

Causative classifications can be used as a diagnostic tool for understanding hydrological model deficiencies. The physical basis of flood forecasting models can also be enhanced by flood‐type‐specific calibration that accounts for different dynamics and processes governing different flood types (Cullmann, Krauße, & Philipp, 2008).

Causative classifications of flood events are also valuable tools for understanding and detecting possible flood changes or nonstationarities, the emergence of new flood types and their implications for estimation (Keller et al., 2018; Turkington et al., 2016). These variations can be analyzed for historic time series (Kampf & Lefsky, 2016; Vormoor et al., 2015) and for future runoff time series generated using projections of climatic models. Modifications of flood types can provide essential information on future flood hazard, and help to detect changes when no significant trends of classical flood characteristics (e.g., peak discharge) are recognizable (Blöschl et al., 2017).

The usefulness of classification depends on the quality of the available flood information and decreases with the length of the flood series. Therefore, for at‐site flood frequency analysis, the gain from using flood‐type‐based mixed distributions has to be first evaluated (Hirschboeck, 2007). For robust application of causative classifications of flood events, especially for flood frequency analysis and design purposes longer time series are required if only MAF series are available. For practical reasons, using shorter, classified flood series might be less beneficial than using long unclassified series sampled in similar fashion, although more research is needed to exactly identify the value of flood event classification in flood frequency estimation. It is, however, advisable to use POT flood sampling to assure that flood samples of every type have a sufficient size (Brunner et al., 2017; Fischer, 2018).

## 7 MISSING INGREDIENTS FOR CAUSATIVE CLASSIFICATIONS OF HYDROLOGICAL PERSPECTIVE

For understanding flood generating mechanisms, it is crucial to consider characteristics that can provide evidence about these processes at the catchment scale. In the following subsections, we discuss ingredients deemed important for this purpose, but that are underrepresented in the existing causative classifications of flood events.

### 7.1 The role of spatiotemporal characteristics of rainfall

Most of the causative classifications of the hydrological perspective use spatially and temporally lumped characteristics as indicators (e.g., total volume of rainfall, maximum precipitation intensity) (Table 2). Rainfall organization and movement within basins is an essential control of flood response and in particular of hydrograph timing (Doswell et al., 1996; Seo, Schmidt, & Sivapalan, 2012; Viglione, Chirico, Woods, & Blöschl, 2010; Zoccatelli, Borga, Viglione, Chirico, & Blöschl, 2011). For certain causative flood types, different space–time characteristics of rainfall are found to be decisive controls for their emergence (Viglione, Chirico, Komma, et al., 2010).

Consideration of spatial moments of rainfall for flood classification might require the availability of data with high spatial and temporal resolutions (radar‐based, Mei et al., 2014). Nevertheless, including space–time rainfall characteristics in a simplified manner might be advantageous for the identification of distinct event types also when just daily data are available (e.g., Keller et al., 2018). Therefore, the use of spatial and temporal characteristics of rainfall as classification criteria can provide additional insight on similarity and dissimilarity of events and result in a more accurate classification.

### 7.2 The role of antecedent wetness state

The wetness state of catchments acts as a linkage between atmospheric and hydrological processes, as well as climatological and hydrological time scales, in the generation of flood events (Hirschboeck et al., 2000). Soil moisture dependent precipitation excess events play a dominant role in controlling seasonality and interannual variability of maximum annual flows (Berghuijs et al., 2016). However, antecedent soil moisture is rarely used as a predictor of flood types in the reviewed classifications. A threshold‐like relationship between event runoff coefficient and soil moisture identified at different spatial scales (Grayson, Western, Chiew, & Blöschl, 1997; Tarasova, Basso, Zink, & Merz, 2018; Zehe & Blöschl, 2004) is an important aspect of catchment behavior linked to the emergence of different runoff generation processes and possibly distinct flood types. The same is true of the temporal variability of the saturated regions, which strongly controls the shape of the flood frequency curve (Rogger, Viglione, Derx, & Blöschl, 2013), especially in mesoscale catchments affected by frontal precipitation events. For determining magnitudes of flash floods, wetness conditions can be even more important than the temporal distribution of storms (Lázaro, Sánchez Navarro, García Gil, & Edo Romero, 2014).

Although the importance of antecedent soil moisture varies regionally (Froidevaux, Schwanbeck, Weingartner, Chevalier, & Martius, 2015), discrepancy between observed extreme precipitation and flood events (Berghuijs et al., 2016) suggests a crucial role of catchment storage state, and calls for an explicit use of soil moisture in classification schemes in order to reach a more comprehensive understanding of flood causation.

### 7.3 The role of routing effects

Properties of flood hydrographs (e.g., peak, volume, duration) are usually assumed to be controlled by the features of triggering precipitation events (Hirschboeck, 1987) and by catchment‐scale runoff generation processes (Merz & Blöschl, 2003). However, routing can attenuate or amplify floods and modify the properties of their hydrographs (Archer, 1989). Routing effects are especially important for large catchments (Falter et al., 2015), where confluences of tributaries might result in considerably different characteristics of event hydrographs downstream (Raynal & Salas, 1987). In fact, even if no existing classification accounts for the routing through channels and floodplains, their effect is frequently discussed (Vorogushyn & Merz, 2013).

The degree of attenuation is highly variable over the range of discharges. A slope break in the flood frequency curve at bankfull discharge mirrors a distinct difference emerging between in‐channel and above‐bankfull events (Archer, 1989). It is therefore advisable to treat those as two distinct flood subsamples (Singh, 1987). When large volume is retained due to dike breach, it may also lead to a slope break in the flood frequency curve and consequentially to considerable flood peak reductions downstream (Apel, Merz, & Thieken, 2009). Finally, Woltemade and Potter (1994) illustrated that floods of intermediate magnitude and high ratio of peak and volume are attenuated by overbank storage the most.

To the best of our knowledge, none of the existing multicriteria approaches considers routing effects for classification of flood events. Therefore, more effort is needed to understand the role of routing processes in steering characteristics of flood events and the possibility of their inclusion into classification schemes.

## 8 CONCLUSIONS AND RECOMMENDATIONS

We examined causative classifications of instrumental and preinstrumental series of flood events that adopt hydroclimatic, hydrological, and hydrograph‐based perspectives. Each of these perspectives has merits and weaknesses. Hydroclimatically defined groups of flood events can be directly linked with the probability of specific weather system type to occur in a certain region, and can be related to global atmospheric processes at longer time scales. They can be useful for addressing flood hazard estimation under nonstationary conditions. However, they neglect flood generation mechanisms at the catchment scale. These mechanisms can be better understood by hydrological classifications, which reflect short‐term rainfall‐delivering mechanisms and their interplay with catchment conditions. Since they only require runoff time series, hydrograph‐based classifications can be easily applied at the location of interest, especially when no other data is available. These application‐oriented approaches offer parsimonious solutions to improve at‐site flood frequency estimates.

So far, no attempt has been made to compare or validate results of different classifications. Hence, there is no agreement about the ingredients of a good classification, let alone a unified method. Most of the reviewed classifications are site‐specific. Although desirable for a specific engineering or managerial task (e.g., linking global scale climatic variability and local scale hydrological responses through process‐sensitive upscaling, Hirschboeck, 2003; improving at‐site flood frequency, Fischer et al., 2016) such frameworks lack transferability in space. Subjective selection of data and grouping approaches may affect classification results. Rigorous uncertainty analyses and testing of the plausibility and applicability would therefore be of great value to enhance the reliability and transferability. These issues should be explicitly addressed in future flood classification studies, in order to bring these methods to their full potential. Ideally, the classification should have the following characteristics:

- **Robust:** using alternative data sources or slightly different thresholds should not result in substantially different classification results;
- **Transferable:** indicators tailored to a specific location should be avoided to allow framework to be applied elsewhere, at least in similar climatic and physiographic conditions;
- **Adaptive:** the framework should have a flexible (e.g., hierarchical) structure that can be adjusted to assist specific hydrological tasks and various practical purposes. This structure should allow for a sizable simplification and a straightforward assessment of its effects on classification results;
- **Consistent:** framework should allow consistent characterization of flood event triggered by the same atmospheric event within river network and account for possible occurrence of mixed‐type flood events generated by several distinct mechanisms or several atmospheric events of disparate origin.

The latter requirement arises from wave‐like behavior of flood events (Diederen, Liu, Gouldby, Diermanse, & Vorogushyn, 2018). A flood event observed at different locations within wave travel time in the river network can be a result of the same atmospheric event. A consistent classification of such events is an inherent feature of large‐scale domain of hydroclimatic classifications. Hydrograph‐based classifications are specific to streamflow gauges and therefore do not guarantee consistent event classification within river network. Hydrological classifications are built on hydrometeorological forcing and catchment states and hence are catchment‐specific, but can be extended by accounting for regional coherency of floods and storms.

Superposition of events at river confluences (Vorogushyn & Merz, 2013) can also mean superposition of types. This is often the case for larger catchments where an event at the outlet is produced by several flood waves generated in different subcatchments by different inducing events. Development of spatially consistent approaches that also account for mixed types is important for comprehensive assessment of flood risk at large spatial scales (Vorogushyn et al., 2018), and for extending their applicability for regional predictions.

The benefit of flood event classification has to be explored beyond local flood frequency analysis, especially for regionalization and flood change detection and attribution. Developing uncertainty analyses and testing procedures for flood type classifications seems necessary to understand their reliability and limitations. More diverse and quantitative multicriteria classification approaches are needed to encompass the wide spectrum of possible flood generation mechanisms and foster a wider use of flood types in hydrological science and practice. If the future frameworks will comply with the above mentioned recommendations, causative classifications will become a powerful tool for deciphering possible changes in flood generation mechanisms and assessing flood hazards in a changing world.

## CONFLICT OF INTEREST

The authors have declared no conflicts of interest for this article.

## RELATED WIREs ARTICLES

Hydrological drought explained

Evolutionary leap in large‐scale flood risk assessment needed
